# Liquid chromatography-tandem mass spectrometry analysis of a ratio-optimized drug pair of *Sophora flavescens* Aiton and *Coptis chinensis* Franch and study on the mechanism of anti-colorectal cancer effect of two alkaloids thereof

**DOI:** 10.3389/fonc.2023.1198467

**Published:** 2023-06-19

**Authors:** Zihan Chen, Yingying Dong, Qiuying Yan, Qin Li, Chengtao Yu, Yueyang Lai, Jiani Tan, Minmin Fan, Changliang Xu, Liu Li, Weixing Shen, Junfei Gu, Haibo Cheng, Dongdong Sun

**Affiliations:** ^1^ School of Integrated Chinese and Western Medicine, Nanjing University of Chinese Medicine, Nanjing, China; ^2^ Jiangsu Collaborative Innovation Center of Traditional Chinese Prevention and Treatment of Tumor Research Center for Theory and Application of Cancer Toxin Pathogenesis, Nanjing, China; ^3^ School of Pharmacy, Nanjing University of Chinese Medicine, Nanjing, China

**Keywords:** *Sophora flavescens* Aiton, *Coptis chinensis* Franch, matrine, berberine, colorectal cancer, intestinal microbiota, RAS signaling pathway, sirt3

## Abstract

The drug pair consisting of *Sophora flavescens* Aiton (*Sophorae flavescentis radix*, Kushen) and *Coptis chinensis* Franch. (*Coptidis rhizoma*, Huanglian), as described in Prescriptions for Universal Relief (Pujifang), is widely used to treat laxation. Matrine and berberine are the major active components of Kushen and Huanglian, respectively. These agents have shown remarkable anti-cancer and anti-inflammatory effects. A mouse model of colorectal cancer was used to determine the most effective combination of Kushen and Huanglian against anti-colorectal cancer. The results showed that the combination of Kushen and Huanglian at a 1:1 ratio exerted the best anti-colorectal cancer effect versus other ratios. Moreover, the anti-colorectal cancer effect and potential mechanism underlying the effects of matrine and berberine were evaluated by the analysis of combination treatment or monotherapy. In addition, the chemical constituents of Kushen and Huanglian were identified and quantified by liquid chromatography-tandem mass spectrometry (LC-MS/MS). A total of 67 chemical components were identified from the Kushen–Huanglian drug pair (water extraction), and the levels of matrine and berberine were 129 and 232 µg/g, respectively. Matrine and berberine reduced the growth of colorectal cancer and relieved the pathological conditions in mice. In addition, the combination of matrine and berberine displayed better anti-colorectal cancer efficacy than monotherapy. Moreover, matrine and berberine reduced the relative abundance of *Bacteroidota* and *Campilobacterota* at phylum level and that of *Helicobacter*, *Lachnospiraceae_*NK4A136_group, *Candidatus_Arthromitus*, norank_f*_Lachnospiraceae*, *Rikenella*, *Odoribacter*, *Streptococcus*, norank_f*_Ruminococcaceae*, and *Anaerotruncus* at the genus level. Western blotting results demonstrated that treatment with matrine and berberine decreased the protein expressions of c-MYC and RAS, whereas it increased that of sirtuin 3 (Sirt3). The findings indicated that the combination of matrine and berberine was more effective in inhibiting colorectal cancer than monotherapy. This beneficial effect might depend on the improvement of intestinal microbiota structure and regulation of the RAS/MEK/ERK-c-MYC-Sirt3 signaling axis.

## Introduction

Colorectal cancer ranks second among malignant tumors in terms of incidence and is the third leading cause of cancer-related mortality (1, 2). Patients with inflammatory bowel disease (IBD) are at a higher risk of developing colorectal cancer. This is because the inflammatory reaction influences the development of tumorigenesis, which is involved in the physiological and pathological reaction process (3).

Recently, an increasing number of research studies focus on the role of intestinal microbiota in the development of colorectal cancer (4–8). The abundance of gut microbiota differs between patients with colorectal cancer and healthy individuals (7, 9, 10). For example, *Bacteroidetes* is more abundant in patients with tubular adenomatous polyps (11). *Helicobacter*, *Lachnospiraceae_*NK4A136_group, *Candidatus_Arthromitus*, norank_f*_Lachnospiraceae*, *Rikenella*, *Odoribacter*, *Streptococcus*, norank_f*_Ruminococcaceae*, and *Anaerotruncus* play indispensable roles in colorectal tumorigenesis and inflammation (12–20). Gut microbiota regulate signaling pathways to promote or delay tumor progression (21–23). Thus, intestinal flora can directly or indirectly regulate tumor-related signaling pathways and affect tumor progression.

The activation and transformation of RAS, an important component of the family of GTPases, have been association with cancer (24). RAS activates the MEK/ERK cascade, thereby altering the transcription of genes relate to the control of extensive intracellular biological mechanisms. Activated RAS recruits RAF kinase, a MAP kinase kinase (MAP3K), to active MEK. In turn, RAF promotes the activation of the effector ERK kinases. Activated ERK phosphorylates several genes involved in cell growth, differentiation, and motility (25), including c-MYC (26). Abnormal c-MYC is involved in genomic instability and tumorigenesis and maintains tumor growth (27). As far back as 1996, the overexpression of c-MYC is considered as a good prognostic factor for survival in colorectal cancer (28). Furthermore, MYC expression improved acetylation-dependent deactivation of succinate dehydrogenase complex flavoprotein subunit A (SDHA) by activating S-phase kinase-associated protein 2-mediated (SKP2-mediated) degradation of Sirt3 deacetylase and tumorigenesis (29).

In recent years, traditional Chinese medicine (TCM) has been used for the prevention and treatment of colorectal carcinoma. It has been shown that TCM inhibits tumorigenesis, improves the therapeutic effect, reduces toxicity, and lowers the risk of recurrence and metastasis (30–34). The effect of TCM on the regulation of intestinal flora is attracting increasing research attention (35–38).

*Sophora flavescens* Aiton (*Sophorae flavescentis radix*, Kushen) and *Coptis chinensis* Franch (*Coptidis rhizoma*, Huanglian) are commonly used in the treatment of intestinal diseases. The earliest use of this drug pair was recorded in the Prescriptions for Universal Relief (Pujifang). In addition, it has been reported that Kushen and Huanglian exert good curative effects in the treatment of tumors (39, 40). Matrine and berberine are two alkaloids contained in Kushen and Huanglian, respectively (41, 42). Matrine prevents colorectal cancer by inhibiting the proliferation, invasion, and metastasis, and inducing the apoptosis of colorectal cancer cells (43–45). Berberine exerts anti-colorectal cancer efficacy by regulating proliferation-related signaling pathway, short-chain fatty acid metabolism, intestinal inflammation, and gut microbiota (46–48). All in all, Kushen and Huanglian and their compounds have certain anti-colorectal cancer effects.

However, the most effective combination of the drug pair Kushen–Huanglian in inhibiting colorectal cancer and the mechanisms underlying the anti-colorectal cancer effect of this combination are currently unclear. Further studies are warranted to investigate whether the oral administration of compounds affects the composition of intestinal flora before it is absorbed into the blood to regulated disease-related molecular mechanisms and play a therapeutic role.

In this study, an orthotopic xenograft mouse model of colorectal cancer was used to investigate the anti-colorectal cancer effect of different combinations of Kushen and Huanglian. LC-MS/MS was performed to identify the chemical constituents of the Kushen and Huanglian. We also examined the mechanism underlying the effects of matrine and berberine on the gut flora of mice with colorectal cancer through sequencing and pathway detection.

## Material and methods

### Chemical and materials

**Figure 1:**
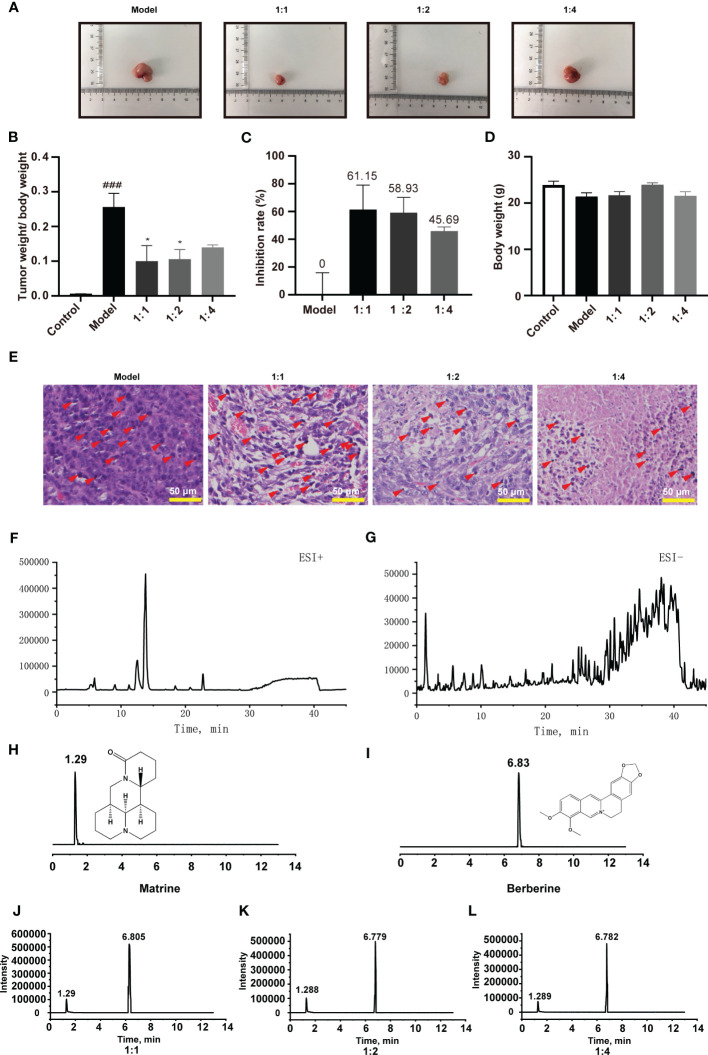
Kushen and Huanglian extract prevents colorectal tumorigenesis in an orthotopic xenograft mouse model of colorectal cancer; chromatogram of Kushen and Huanglian (1:1) extract. **(A)** Representative image of tumor tissue from model mice. **(B)** Tumor weight/body weight of mice (*n* = 6, ^###^ *p* < 0.001 compared with Control group, *p < 0.05 compared with Model group). **(C)** Inhibitory rate of Kushen and Huanglian extract on colorectal cancer in model mice (*n* = 6). **(D)** Body weight of mice (*n* = 6). **(E)** Representative image of HE-stanned tumor tissue obtained from model mice. Scale bar: 50 μm. **(F, G)** Total iron chromatogram of Kushen and Huanglian (1:1) extract in ESI^+^ **(F)** and ESI^−^ **(G)** mode. **(H, I)** MRM chromatogram of matrine **(H)** and berberine **(I)**. **(J–L)** MRM chromatogram of matrine and berberine in Kushen and Huanglian extract [**(J)** 1:1; **(K)** 1:2; **(L)** 1:4)]. Red arrows indicate nuclear pyknosis.

Matrine (purity≥98%, Cat. No. B20679) and berberine (purity≥ 98%, Cat. No. B21379) were purchased from Shanghai Yuanye Bio-Technology Co., Ltd. (Shanghai, China) (**Figures 1H, I**). The hematoxylin–eosin (HE) staining kit (Cat. No. G1120) was obtained from Solarbio (Beijing, China). Antibodies (c-MYC (Cat. No. 18583), phosphorylated-MEK [p-MEK] (Cat. No. 3958), MEK (Cat. No. 2352), p-ERK (Cat. No. 9106), ERK (Cat. No. 9102), Sirt3 (Cat. No. 2627), RAS (Cat. No. 67648), and glyceraldehyde-3-phosphate dehydrogenase [GAPDH] (Cat. No. 5174) were purchased from Cell Signaling Technology (Beverly, MA, USA). Horseradish-peroxidase (HRP)-labeled secondary antibodies (Cat. Nos. ab205718 and ab6789) were obtained from Abcam Plc (Cambridge, UK). Fufang Banmao capsules were produced by Guizhou Ebay Pharmaceutical Corporate Co. Ltd. (Guizhou, China). Acetonitrile gradient grade (Cat. No. 1.06007) for liquid chromatography were purchased from Merck KGaA (Darmstadt, Germany). Kushen (from Chifeng, Inner Mongolia) and Huanglian (from Dazhou, Sichuan province) were purchased from Jiangsu Province Hospital of Chinese Medicine (Nanjing, China).

### Water extraction

For water extraction, Kushen and Huanglian were soaked in water (10× volume) for 30 min at 100°C. This process was repeated in a reduced volume of water (8×). The two extracts were combined and concentrated to 1.6 g crude drug/ml at 60°C. The extraction samples containing 1 g crude drug were dissolved in methanol solution (30 ml) and sonicated for 30 min. Next, the extractions were filtered using a filter unit (pore size, 0.45 μm) and diluted to an appropriate concentration for LC/MS analysis.

### LC/MS analysis

An AB Sciex 5600 Triple ultra-performance liquid chromatography time-of-flight tandem mass spectrometry (UPLC-TOF-MS/MS) spectrometer was utilized to analyze the extracts of Kushen and Huanglian. A Waters Acquity UPLC HSS T3 column (1.8 µm, 2.1 mm × 50 mm) was used for the chromatographic separation. The LC eluents were acetonitrile (A) and deionized water with 0.1% formic acid (B). The gradient used was as follows: 0–1 min, 5% B; 1–33 min, 5%–95%B; 33–38 min, 95% B; 38–40 min, 95%–5% B; and 40–45 min, 5% B. The injection volume was 2 µl, and the flowrate was 0.3 ml/min.

The AB Sciex 5500 Triple UPLC-TQ-MS/MS was used for the quantitative analysis of Kushen and Huanglian extractions. A total of 10 chemical components were identified and quantified by using AB Sciex 5500 Triple Quad MS platform with multiple reaction monitoring (MRM). A Waters Acquity UPLC HSS T3 column (1.8 µm, 2.1 mm × 50 mm) was used for the chromatographic separation. The LC eluents were acetonitrile (A) and deionized water with 0.1% formic acid (B). The gradient used was as follows: 0–1 min, 15% B; 1–5 min, 15%–50%B; 5–7 min, 50% B; 7–10 min, 50%–15% B; and 10–13 min, 15% B. The injection volume was 5 µl, and the flowrate was 0.3 ml/min.

### Standard solutions preparation

Matrine and berberine were accurately weighed and dissolved in methanol to obtain stock solutions at the concentration of 600 μg/ml. Mixed stock standard solution was prepared in methanol, and the concentrations of matrine and berberine in mix stock solution were 600 ng/ml. Then, the mixed standard stock solutions were diluted to seven concentrations for calibration curves. The linear range of matrine is 0.5–600 ng/ml, and berberine is 1–600 ng/ml.

### Animal experiment

The protocol for animal experiments was approved by the Nanjing University of Chinese Medicine Institutional Animal Care and Use Committee (approval number of the Ethics Committee on Animal Experimentation: 202012A010). All animal experiments were carried out under the guidelines approved by the Institutional Animal Care and Use Committee of Nanjing University of Chinese Medicine. CT26.WT cells (1 × 10^6^ cells) were injected into subcutis of five BALB/c nude mice. When the tumor volume reached approximately 100 mm^3^, the mice were anesthetized, and the tumor tissues were extracted and cut into squares (~1 mm^3^). A blade was used to scratch the exposed proximal colon serosa of BALB/c nude mice, and tumor tissue fragments (1 mm^3^) were attached on the damaged proximal colon serosa and covered with tissue adhesive (10 μl). The adessive was allowed to fully solidify for an additional 60 s; thereafter, the peritoneum and skin were sutured with a nylon suture (Cat. No. 220103, obtained from Ningbo Medical Needle Co., Ltd., Ningbo, China).

The mice that had undergone surgery were classified into 11 groups (six for each group): model group; Fufang Banmao Capsules group (positive group); Kushen and Huanglian (1:1) group (1:1; 0.6 g/kg/day); Kushen and Huanglian (1:2) group (1:2; 0.6 g/kg/day); Kushen and Huanglian (1:4) group (1:4; 0.6 g/kg/day); low-matrine (MT-Low) group (15 mg/kg/day); high-matrine (MT-High) group (30 mg/kg/day); low-berberine (BBR-Low) group (30 mg/kg/day); high-berberine (BBR-High) group (200 mg/kg/day); matrine and low-berberine (MT+BBR-Low) group (100 mg/kg/day; ratio of matrine and berberine was 1:2); and matrine and high-berberine (MT+BBR-High) group (200 mg/kg/day; ratio of matrine and berberine was 1:2). Six mice underwent surgery without the use of tissue adhesive (control group). Mice in all groups received the extract in normal saline by gavage.

Mice were treated for 21 days. Thereafter, they were anesthetized for tumor extraction. Tumor tissues were collected, divided into two pieces, and subjected to Western blotting, histology, and immunohistochemistry analyses.

### HE staining and immunohistochemistry

For histopathological evaluation, tumor tissue sections were stained with HE solution. Thereafter, the sections were observed under an inverted microscope to identify alterations.

For immunohistochemistry, sections were incubated with a primary antibody against Sirt3 overnight at 4°C. Next, they were washed using phosphate-buffered saline (PBS) buffer and incubated with HRP-labeled secondary antibody for 1 h at room temperature. A DAB kit was used for staining. The analysis of cells that exhibited positivity for Sirt3 was performed using the image analysis software ImageJ. Representative images were captured from six independent samples.

### 16S Ribosomal RNA gene sequencing and bioinformatics analysis

DNA of intestinal flora was extracted from fecal samples frozen at −80°C (n = 6 per group). The samples were processed and analyzed by Shanghai Majorbiao Bio-Pharm Technology Co. Ltd. (Shanghai, China). The V3–V4 hypervariable region of bacterial 16S rRNA was amplified using polymerase chain reaction (PCR) with barcode-indexed primers 319F (5′-ACTCCTACGGGAGGCAGCAG-3′) and 806R (5′-GGACTACHVGGTTTCTCATAT-3′). Gel extraction was used for the purification of amplicon. Following the quantification of amplicons, library preparation and sequencing were performed using a TruSeq Nano DNA LT Library Prep Kit. Operational taxonomic units (OTUs) with over 97% sequence similarity were used for data analyzed, which was performed on the Majorbio Cloud Platform (www.majorbio.com). The diversity of the samples is presented as alpha diversity, while OUT, genus species, and abundance were used to calculate the Chao and Shannon diversity indices. The Chao index denoted microbiota richness in the sample, while the Shannon index represented community diversity. Furthermore, differences in the diversity of intestinal microbiota between groups were determined using principal coordinates analysis (PCOA) and partial least squares discriminant analysis (PLS-DA).

### Western blot

Radioimmunoprecipitation assay (RIPA) buffer (Cat. No. P0013C, obtained from Beyotime Biotechnology, Shanghai, China) containing protease and phosphorylase inhibitor cocktails (Cat. No. P1046, obtained from Beyotime Biotechnology, Shanghai, China) was used to obtain protein from frozen tissues. The determination of protein content was performed by using BCA protein assay kit (Cat. No. P0010, obtained from Beyotime Biotechnology, Shanghai, China). Sodium dodecyl-sulfate polyacrylamide gel electrophoresis (SDS-PAGE) gel was used to separate proteins, which were subsequently transferred onto a polyvinylidene fluoride membrane. Next, the membranes were incubated with 5% skimmed milk for 2 h to block non-specific binding and incubated with primary antibodies overnight at 4°C. The next day, membranes were washed with TBST buffer and incubated with secondary antibody for 2 h at room temperature. The immunoreactivity was revealed by using an ECL chromogenic substrate, visualized through an imaging system, and quantitatively analyzed by using ImageJ software.

### Statistical analysis

Values were presented as the means ± standard error of the mean. Student’s *t*-test and one-way ANOVA were used for statistical analysis, and *p-*values < 0.05 denoted statistically significant differences. The graph was drawn by using GraphPad Prism 9 and Majorbio Cloud Platform.

## Results

### Kushen and Huanglian extract prevented colorectal tumorigenesis

The tumor tissue weight, body weight, and the tumor inhibitory rate were calculated. As shown in **Figures 1A–C**, Kushen–Huanglian extract significantly reduced the ratio of tumor tissue weight to body weight of the model mice. Notably, the extraction at a 1:1 ratio was the most effective among the preparations.

The extract of Kushen and Huanglian did not exert an effect on the body weight of the model mice (**Figure 1D**), suggesting that there was no physical sign of poor condition in any of the experimental mice. According to the results of HE staining, treatment with Kushen and Huanglian effectively reduced the histological damage compared with control (**Figure 1E**). These results revealed that the combination of Kushen and Huanglian at a1:1 ratio showed the best inhibitory effect on colorectal tumorigenesis in mice with cancer versus other preparations.

### Qualitative and quantitative analysis of Kushen and Huanglian extract

UPLC-TOF-electrospray ionization-MS/MS (UPLC-TOF-ESI-MS/MS) was used to analyzed the constituents of Kushen and Huanglian extract (1:1 ratio). The total ion chromatogram (TIC) (shown in **Figures 1F, G**) displays the major peaks, which were studied using positive and negative ESI modes. A total of 67 compounds were identified according to a previous report (**Table 1**).

**Table 1:** Identified chemical compounds in the extract from Kushen and Huanglian.

NO	Compound	Molecular formula	*t*_R_ (min)	Measured molecular weight	Theoretical molecular weight	Fragment ion	Tolerance (ppm)
1	N-Methylcytisine	C_12_H_16_N_2_O	1.4844	205.1337	205.1335	149,108,58	0.84
2	9α-Hydroxysophoramine	C_15_H_20_N_2_O_2_	1.4878	261.1601	261.1598	243,177,150	1.26
3	7α- Hydroxysophoramine	C_15_H_20_N_2_O_2_	1.4878	261.1601	261.1598	243,177,150	1.26
4	Anagyrine	C_15_H_20_N_2_O	1.5625	245.1647	245.1654	148	2.80
5	Lupanine	C_15_H_24_N_2_O	1.5642	249.1964	249.1961	166,136	1.18
6	Matrine	C_15_H_24_N_2_O	1.6116	249.1961	249.1961	176,148	0.00
7	Isosophocarpine	C_15_H_22_N_2_O	2.0622	247.1814	247.1810	179,150,148,136	1.51
8	Sophocarpine	C_15_H_22_N_2_O	2.3227	247.1809	247.1805	179,150,136	1.69
9	5,6-Dehydrolupanine	C_15_H_22_N_2_O	2.3227	247.1809	247.1810	176,150,136	0.40
10	Baptifoline	C_13_H_22_N_2_O_2_	2.5579	261.1600	261.1598	243,164,114	0.71
11	9α-Hydroxymatrine	C_15_H_24_N_2_O_2_	4.6859	265.1907	265.1916	247,150,148,112	3.00
12	Oxymatrine	C_15_H_24_N_2_O_2_	4.7889	265.1908	265.1911	247,205,148	1.10
13	5α-Hydroxymatrine	C_15_H_24_N_2_O_2_	4.7889	265.1908	265.1916	247,150,148,112	3.00
14	14β-Hydroxymatrine	C_15_H_24_N_2_O_2_	4.7889	265.1908	265.1916	247,150	3.00
15	Sophoridine	C_15_H_24_N_2_O	4.8957	249.1965	249.1961	150	1.74
16	Isomatrine	C_15_H_24_N_2_O	4.9702	249.1968	249.1967	176,150,148	0.21
17	Cis-caffeic acid	C_9_H_8_O_4_	5.6607	181.0495	181.0495	181,163,135,117,93,65	0.10
18	Mamanine	C_15_H_22_N_2_O_2_	5.7132	263.1756	263.1754	231	0.67
19	Oxysophocarpine	C_15_H_22_N_2_O_2_	5.7860	263.1758	263.1754	245,150	1.66
20	Sophoranol	C_15_H_24_N_2_O_2_	5.8503	265.1913	265.1916	247,205	1.20
21	7,11-Dehydromatrine	C_15_H_22_N_2_O	6.6567	247.1809	247.1810	176,148	0.40
22	Sophoranol N-oxide	C_15_H_24_N_2_O_3_	8.1026	281.1863	281.1860	263,243,149	1.13
23	13-Hydroxycolumbamine	C_20_H_20_NO_5_	8.6227	354.1328	354.1326	339,324,296,306,278	0.45
24	Magnoflorine	C_20_H_24_NO_4_	9.0748	342.1705	342.1705	297,265,237	0.07
25	Norisocorydine	C_19_H_22_NO_4_	9.2687	328.1547	328.1543	313,298	1.24
26	Tetradehydroscoulerine	C_19_H_16_NO_4_	9.6160	322.1068	322.1068	307,294,279	0.00
27	Tetradehydrocheilanthifolinium	C_19_H_16_NO_4_	9.6160	322.1068	322.1068	307,294,279	0.00
28	Lycoranine B	C_18_H_13_NO_4_	9.9450	308.0917	308.0918	280,265,250	0.40
29	N-methlylcorydalmine	C_21_H_26_NO_4_	10.5913	356.1858	356.1857	206	0.27
30	Menisperine	C_21_H_26_NO_4_	10.5928	356.1859	356.1853	311, 296	1.75
31	Demethyleneberberine	C_19_H_18_NO_4_	10.8732	324.1228	324.1231	309,294,266;280	1.00
32	Stecepharine	C_21_H_26_NO_5_	10.9242	372.1804	372.1806	222,207,189	0.60
33	Beberrubine	C_19_H_16_NO_2_	11.0160	322.1080	322.1079	322,307,279,250	0.38
34	Groenlandicine	C_19_H_16_NO_4_	11.2061	322.1071	322.1079	307,279	2.40
35	Thalifendine	C_19_H_16_NO_4_	11.2061	322.1071	322.1072	307,294,279	0.20
36	Columbamine	C_20_H_20_NO_4_	11.2668	338.1402	338.1391	322,294;308,280,265	3.23
37	Noroxyhydrastinine	C_10_H_9_NO_3_	11.5192	192.0656	192.0655	192,174,163,192	0.37
38	13-Methyljatrorrhizine	C_20_H_18_NO_5_	11.5208	352.1184	352.1184	337,322,294,336,308	0.13
39	Oxyberberine	C_20_H_17_NO_5_	11.5446	352.1179	352.1180	337,322,308,294	0.20
40	13-Hydroxyberberine	C_20_H_18_NO_5_	11.5651	352.1182	352.1180	337,336,308,322,318	0.43
41	Protopine	C_20_H_19_NO_5_	11.8251	354.1339	354.1336	354,339,324,310	0.95
42	Tetrahydroberberine	C_20_H_21_NO_4_	12.2755	338.1383	338.1387	338,323,295	1.10
43	Coptisine	C_19_H_14_NO_4_	12.6173	320.0923	320.0923	318,290,262,249	0.00
44	Palmatine	C_20_H_18_NO_5_	12.9400	352.1529	352.1535	337,322,308,294	1.80
45	13-Methylberberine chloride	C_21_H_20_NO_4_	13.1289	350.1392	350.1392	147,176	0.10
46	Worenine	C_20_H_15_NO_4_	13.4120	334.1082	334.1079	334,319,304,290,277	0.83
47	Epiberberine	C_20_H_18_NO_4_	13.7474	336.1238	336.1227	320,292	3.25
48	Berberine	C_20_H_18_NO_4_	13.8362	336.1238	336.1228	336,320,290,278	2.90
49	Linarin	C_28_H_32_O_14_	14.0601	593.1843	593.1838	593,447,285	0.89
50	Daidzein	C_15_H_10_O_4_	14.3132	255.0663	255.0652	227	4.40
51	13-Methylpalmatine	C_22_H_24_NO_4_	14.3165	366.1705	366.1700	351,334,322,308,306	1.35
52	13-Methylberberine	C_20_H_17_NO_4_	13.8362	336.1238	336.1230	334,322	0.40
53	Calycosin	C_16_H_12_O_5_	14.9714	285.0752	285.0758	270,253,225	2.00
54	Cytisine	C_11_H_14_N_2_O	16.1558	191.1173	191.1179	150,148	2.90
55	Formononetin	C_16_H_12_O_4_	18.3435	269.0809	269.0811	254,213,137,118	0.70
56	8-Oxocoptisine	C_19_H_13_NO_5_	20.5454	336.0868	336.0867	336,308,293,278	0.54
57	Kuraramine	C_12_H_18_N_2_O_2_	36.4231	223.1439	223.1441	191,162,114	0.95
58	Anagyrine	C_15_H_20_N_2_O	1.46215	245.1651	245.1648	148,118,98	1.11
59	Danshensu	C_9_H_10_O_5_	5.9774	197.0469	197.0458	197,179,135,123	5.61
60	Vanillic acid	C_8_H_8_O_4_	6.1849	167.0352	167.0350	167,152,108	1.06
61	3,4-Dihydroxybenzoic acid	C_7_H_6_O_4_	6.4494	153.0198	153.0194	153,109,91,80	2.64
62	Phellodendrin	C_20_H_24_NO_4_	9.0036	340.1557	340.1543	340,325,310,282,267	4.15
63	Dehydrocheilanthifoline	C_19_H_15_NO_4_	11.5361	321.0995	321.0995	321, 193, 178, 134	0.05
64	Canadine	C_20_H_21_NO_4_	11.2668	338.1402	338.1386	338,323,308,293,264	4.71
65	3-O-Feruloylquinic acid	C_17_H_20_O_9_	13.1451	367.1026	367.1029	191,173	0.80
66	5-O-Feruloylquinic acid	C_17_H_20_O_9_	13.1451	367.1026	367.1029	191	0.80
67	4-O-Feruloylquinic acid	C_17_H_20_O_9_	13.1451	367.1026	367.1029	191,173	0.80

UPLC-triple quadruple-MS/MS (UPLC-TQ-MS/MS) was used to quantitatively analyze matrine and berberine in the Kushen and Huanglian extract. The peaks of matrine and berberine are shown in **Figures 1H–L**. The levels of matrine and berberine were calculated based on their calibration curves. The results are shown in **Table 2**. The levels of matrine and berberine were 129 µg/g (0.129‰ of the herb) and 232 µg/g (0.232‰ of the herb). The ratio was approximately 1:2, which was used in the subsequent experiments.

**Table 2:** The contents of matrine and berberine in the extract from Kushen and Huanglian.

Compound	*t*_R_ (min)	Regression equation	R^2^	Linear rang (ng/ml)	Content (µg/g)
Matrine	1.29	Y = 34340*X − 86061	0.999	0.5–600	129.47 ± 9.82
Berberine	6.83	Y = 97034*X + 2117352	0.999	1–600	232.87 ± 89.94

### Matrine and berberine prevented colorectal tumorigenesis

**Figure 2:**
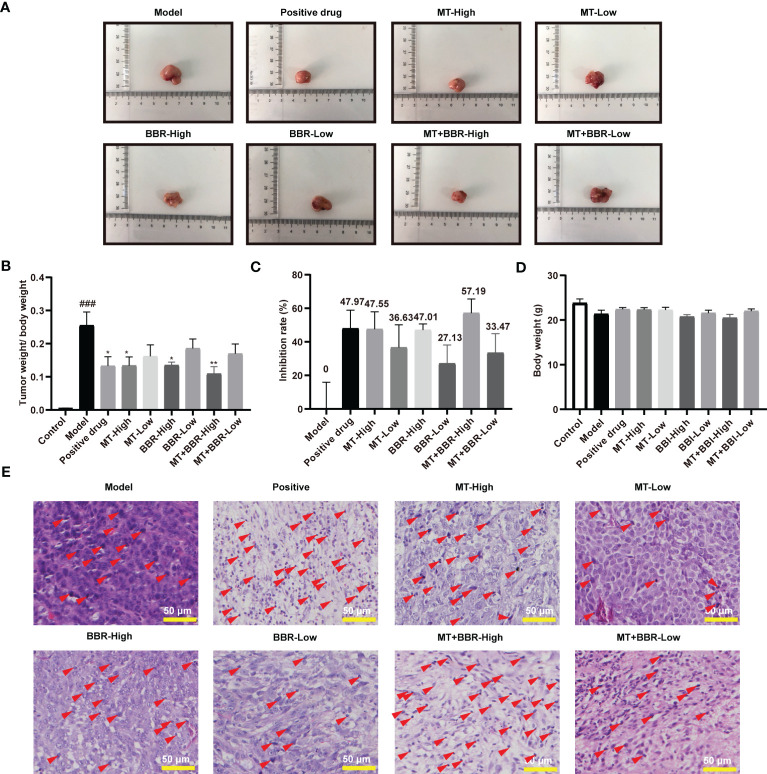
Matrine and berberine prevent colorectal tumorigenesis in an orthotopic xenograft model of colorectal cancer. **(A)** Representative image of tumor tissue obtained from model mice. **(B)** Tumor weight/body weight of mice (*n* = 6, ^###^ *p* < 0.001 compared with Control group, *p < 0.05, ** *p* < 0.01 compared with Model group). **(C)** Inhibitory rate of matrine, berberine, and positive drug on colorectal cancer in model mice (*n* = 6). **(D)** Body weight of mice (*n* = 6). **(E)** Representative image of HE-stained tumor tissue from model mice. Scale bar: 50 μm. Red arrows indicate nuclear pyknosis.

The tumor weights of mice in the MT-low group, BBR-low group, and MT+BBR-low group mice were decreased compared with that of mice in the control group; however, the difference was not statistically significant. In contrast, the tumor weight of MT-high group, BBR-high group, and MT+BBR-high group was remarkably declined (**Figures 2A, B**). The inhibitory rate in each group is displayed in **Figure 2C**. Of note, differences in the body weight between groups were not statistically significant (**Figure 2D**). Nevertheless, treatment with matrine and berberine reduced the weight of tumor tissue in the model mice.

Subsequently, pathological changes in tumor tissues were detected by HE staining. In the model group, the morphology of tumor tissue was obviously atypical and dense and was characterized by irregular arrangements. Compared with the model group, treatment with matrine and berberine (particularly at high dosage) effectively reduced the histological damage. As shown in **Figure 2E**, the morphological alterations demonstrated that, compared with the model group, positive drug, and treatment with matrine and berberine significantly reduced tumor cell density in the tumors. These results revealed that matrine and berberine could inhibit colorectal tumorigenesis in model mice.

### Matrine and berberine improved the intestinal microbiota structure

**Figure 3:**
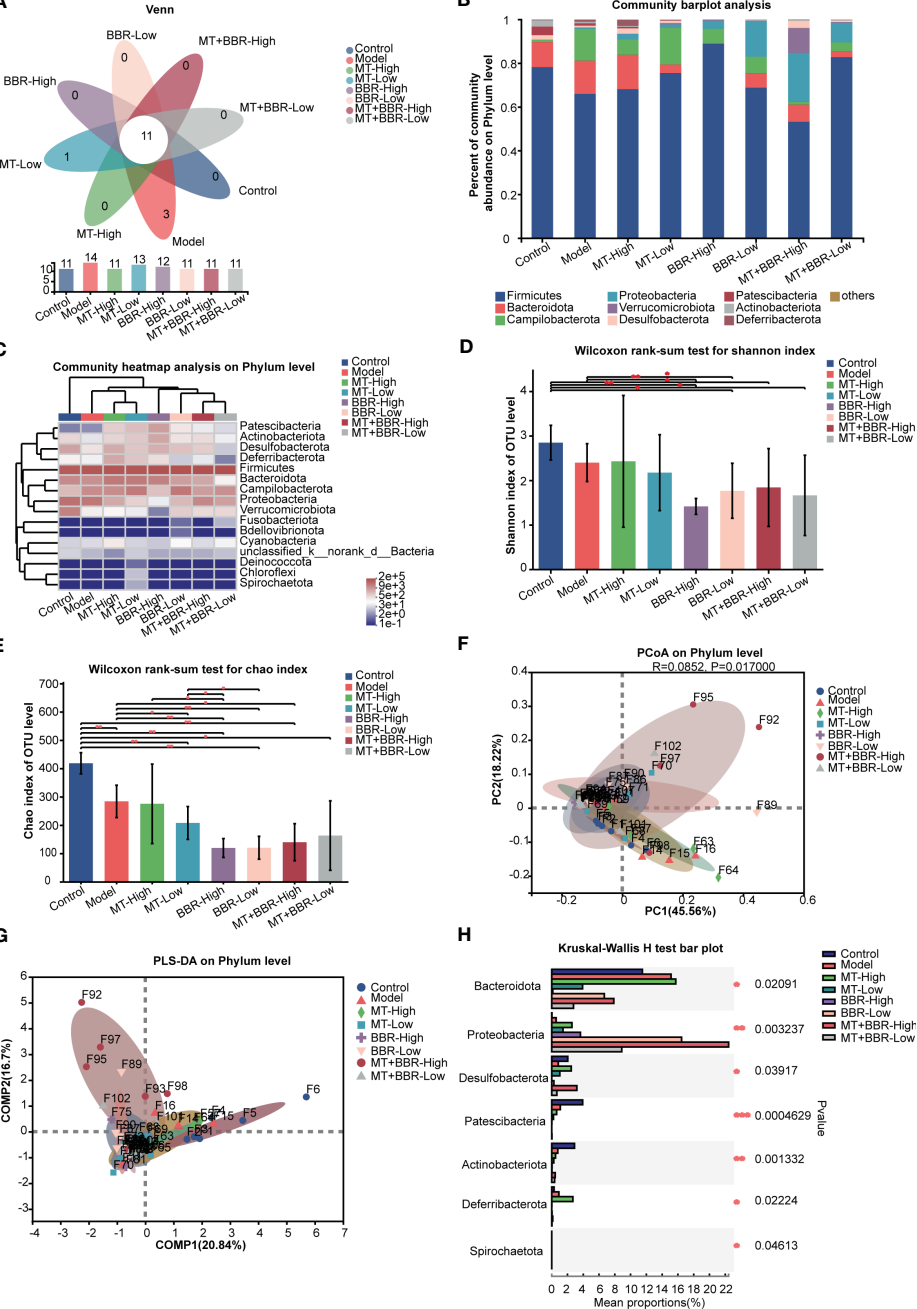
Matrine and berberine regulated the species diversity of intestinal microbiota in mice at the phylum level. **(A)** Venn diagram summarizing the numbers of common and unique phyla. **(B)** Relative abundance of the top 10 phyla classified as colorectal microbiota constituents. **(C)** Abundance of gut microbiota species at the phylum level in each group represented by clustering heatmap. **(D, E)** Alpha diversity of the gut microflora in mouse fecal at the phylum level, represented by the Shannon **(D)** and Chao **(E)** indices. **(F, G)** Matrine- and berberine-mediated impact on bacterial composition in model mice at the phylum level, represented by PCoA **(F)** and PLS-DA **(G)** analysis **(H)** Bar plot of compositional differences in the gut microbiome between groups at the phylum level, calculated by the Wilcoxon rank-sum test. *n* =6, *p < 0.05, **p < 0.001, ***p < 0.001.

**Figure 4:**
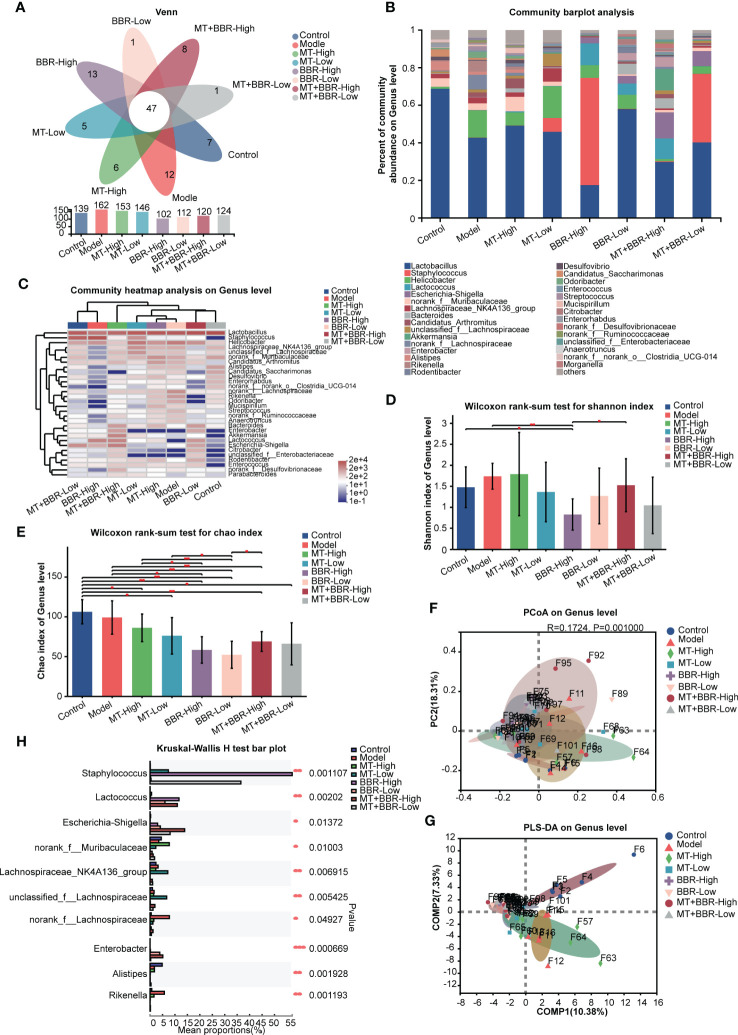
Matrine and berberine regulated the species diversity of intestinal microbiota in mice at the genus level. **(A)** Venn diagram summarizing the numbers of common and unique genera. **(B)** Relative abundance of the top genera classified as colorectal microbiota constituents. **(C)** Abundance of gut microbiota species in each group at the genus level, represented by clustering heatmap. **(D, E)** Alpha diversity of the gut microflora in mouse fecal samples at the genus level, represented by Shannon **(D)** and Chao **(E)** indices. **(F, G)** Matrine- and berberine-mediated impact on bacterial composition in model mice at the genus level, represented by PCoA **(F)** and PLS-DA **(G)** analysis. **(H)** Bar plot of compositional differences in the gut microbiome between groups at the genus level, calculated using the Wilcoxon rank-sum test. *n* =6, *p < 0.05, **p < 0.001, ***p < 0.001.

Thereafter, we sought to characterize the effects of treatment with matrine and berberine on the composition of intestinal microbiome through analysis of bacterial 16S rRNA compositions. The Venn diagram indicated that the number of OTUs was higher at the phylum and genus levels in the model group compared with the control or matrine and berberine treatment groups (**Figures 3A**, **4A**). Higher Shannon index values represented higher species diversity in the sample. The results demonstrated that treatment with matrine and berberine significantly reduced the Shannon and Chao indices in the model mice (**Figures 3D, E**, **4D, E**). Moreover, the abundances of genera in each group were visualized on a heatmap, displaying trends at the phylum and genus levels. In addition, differences in the composition of the intestinal microbiome at the phylum and genus levels were recorded in the individual treatment groups (**Figures 3C**, **4C**). The abundance diversity histogram of the top 10 phylum structure demonstrated that the *Bacteroidota* and *Campilobacterota* (i.e., the predominant flora in the samples) presented a difference between the model and matrine and berberine treatment groups (**Figure 3B**). Further study demonstrated that treatment with matrine and berberine reduced the abundance of *Helicobacter*, *Lachnospiraceae_*NK4A136_group, *Candidatus_Arthromitus*, norank_f*_Lachnospiraceae*, *Rikenella*, *Odoribacter*, *Streptococcus*, norank_f*_Ruminococcaceae*, and *Anaerotruncus* at the genus level (**Figure 4B**).

These results revealed that the species richness of intestinal microbiota in the orthotopic xenograft colorectal cancer model mice was observably downregulated following the administration of matrine and berberine.

Notably, different groups exhibited distinct bacterial composition (**Figures 3F, G**, **4F, G**). Alterations at the phylum and genus levels were also assessed. The results revealed statistically significant differences in seven phyla (i.e., *Bacteroidota*, *Proteobacteria*, *Desulfobacterota*, *Patescibacteria*, *Actinobacteriota*, *Deferribacterota*, and *Spirochaetota*) and 10 genera (i.e., *Staphylococcus*, *Lactococcus*, *Esherichia-Shigella*, norank_f_*Muribaculaceae*, *Lachnospiraceae*_NK4A136_group, unclassified_f_*Lachnospiraceae*, norank_f_*Lachnospiraceae*, *Enterobacter*, *Alistipes*, and *Rikenella*) (**Figures 3H**, **4H**). The abundance of *Proteobacteria* in the matrine- and berberine-treated groups was higher than that recorded in the model group. In contrast, the abundance of the other six phyla was lower than that in the model group.

### Matrine and berberine decreased c-MYC expression, inhibited RAS signaling pathway, and increased Sirt3 expression

**Figure 5:**
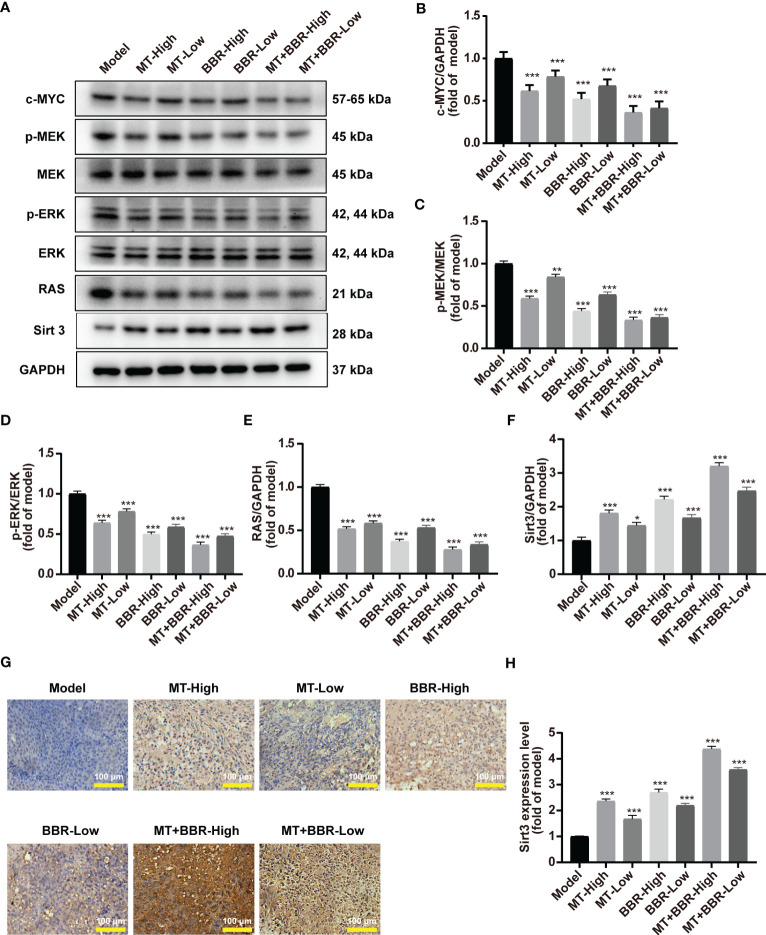
Matrine and berberine decreased c-MYC expression, inhibited RAS signaling, and increased SIRT3 expression in model mice. **(A–F)** Representative Western blotting with quantification results for proteins present in tumor tissue obtained from mice (*n* = 6, ***p < 0.001). **(G, H)** Representative immunohistochemistry images with quantification results for Sirt3 in tumor tissue obtained from mice (*n* = 6, ***p < 0.001). Scale bar: 100 μm. *p<0.05, **p<0.01.

The Western blot analysis demonstrated that treatment with matrine and berberine decreased the expression of transcription factor c-MYC *in vivo* (**Figures 5A, B**).

The efficacy of matrine and berberine to regulate the RAS/MEK/ERK signaling pathway was also inquired *in vivo*. The levels of RAS, MEK, p-MEK, ERK, and p-ERK in tumor tissues were detected through Western blot. The levels of RAS, p-MEK, and p-ERK in the neoplasm tissue obtained from model mice were significantly reduced after treatment with matrine and berberine (**Figures 5A, C–F**). Furthermore, our results suggested that treatment with matrine and berberine upregulated the expression of Sirt3 in tumorous tissue (**Figures 5A, F–H**).

## Discussion

Oral preparation with TCM has a complex composition. The active components of such preparations were absorbed and distributed through the gastrointestinal tract to reach the site of action. Before being absorbed, the compounds could perturb the composition of the gastrointestinal flora (15, 20).

In TCM, the combination of Kushen and Huanglian is used for the treatment of intestinal disease. The major active compounds from Kushen and Huanglian also reduce the inflammation action and exhibit anti-colorectal cancer efficacy (49–54). Different drug ratios can affect the effectiveness of the herbs. According to the record of the Pujifang, the proportion of Kushen and Huanglian was 1:2. In this study, we evaluated Kushen and Huanglian at 1:1, 1:2, and 1:4 ratios to determine the combination with the best anti-tumor effect. We found that the combination at a 1:1 ratio exhibited the most potent anti-colorectal cancer effect. The chemical constituents of this preparation were detected using of UPLC-TOF-ESI-MS/MS. A total of 67 compounds were identified according to literature data. Further analysis showed that the contents of matrine and berberine were 129.47 and 232.87 µg/g, respectively. The ratio of matrine and berberine was approximately 1:2. Thus, we used matrine and berberine at this ratio to evaluate their effect against colorectal cancer. According to the literature (55–57), 15 and 30 mg/kg matrine and 100 and 200 mg/kg berberine were used to mice. In the combination of two compounds group, the total dosage of two compounds were 100 and 200 mg/kg, with the ratio of matrine to berberine of 1:2. We showed that the combination of matrine and berberine exerted a better anti-colorectal cancer effect in the orthotopic xenograft colorectal model mice than monotherapy.

The gut microbiota is a key component of the colorectal cancer microenvironment. Intestinal microecology disorders are strongly associated with the development and progression of colorectal cancer (58). In our animal experiments, the mice were treated by oral gavage. Before the drug was absorbed into the blood in the gastrointestinal tract, the drug may affect the composition of the gastrointestinal flora before it is absorbed. Therefore, we analyzed the mouse fecal gut flora by 16S rRNA sequencing. Treatment with matrine and berberine treatment significantly reduced the diversity of intestinal microbiota in tumor-bearing mice. According to the literatures, *Proteobacteria* abnormal expansion is usually considered as a signature of dysbiosis in gut microbiota. Irinotecan is widely used in the treatment of colorectal cancer; it can also increase the level of *Proteobacteria* and cause intestinal mucositis with diarrhea (59). The combined matrine and berberine increased the abundance of *Proteobacteria*, suggesting that they might be causing intestinal mucositis with diarrhea, which should be noted when using the combined matrine and berberine in the treatment of colorectal cancer. The abundance of *Bacteroidota* and *Campilobacterota* was positive related to colorectal cancer (13, 60). Furthermore, our experiment showed that matrine and berberine decreased the abundance of *Bacteroidota* and *Campilobacterota*. According to the previous research, *Helicobacter pylori* (an important member of *Campilobacterota*) could encode *CagA* gene to activate the RAS/MEK/ERK signaling pathway (61). RAS/MEK/ERK is one of the most dysregulated signaling pathway in cancer. Extensive research has been conducted on the development of inhibitors targeting the RAS/RAF-MEK-ERK/MAPK signaling pathway (25). Due to its active compounds, the Huanglian and Kushen drug pair exerts an anti-cancer effect by regulating the RAS/ERK/MEK-related signaling pathway (62–64). Thus, we detected the effect of matrine and berberine on the expression of proteins linked to the RAS/MEK/ERK signaling axis in tumor tissues obtained from mice. Matrine and berberine significantly inhibited the RAS/ERK/MEK signaling pathway by decreasing the phosphorylation levels of MEK and ERK and expression of RAS. Furthermore, matrine and berberine decreased the level of c-MYC protein, which could improve acetylation-dependent deactivation of SDHA by activating SKP2-mediated degradation of Sirt3 deacetylase and tumorigenesis. Moreover, our results demonstrated that matrine and berberine improved the expression of Sirt3, which could inhibit growth of tumor cells (65). Therefore, matrine and berberine could inhibit MYC-induced tumorigenesis by regulating degradation of Sirt3 deacetylase. These findings suggested that matrine and berberine exert its anti-colorectal cancer effect by regulating the RAS/MEK/ERK-c-MYC-Sirt3 signaling axis.

**Figure 6:**
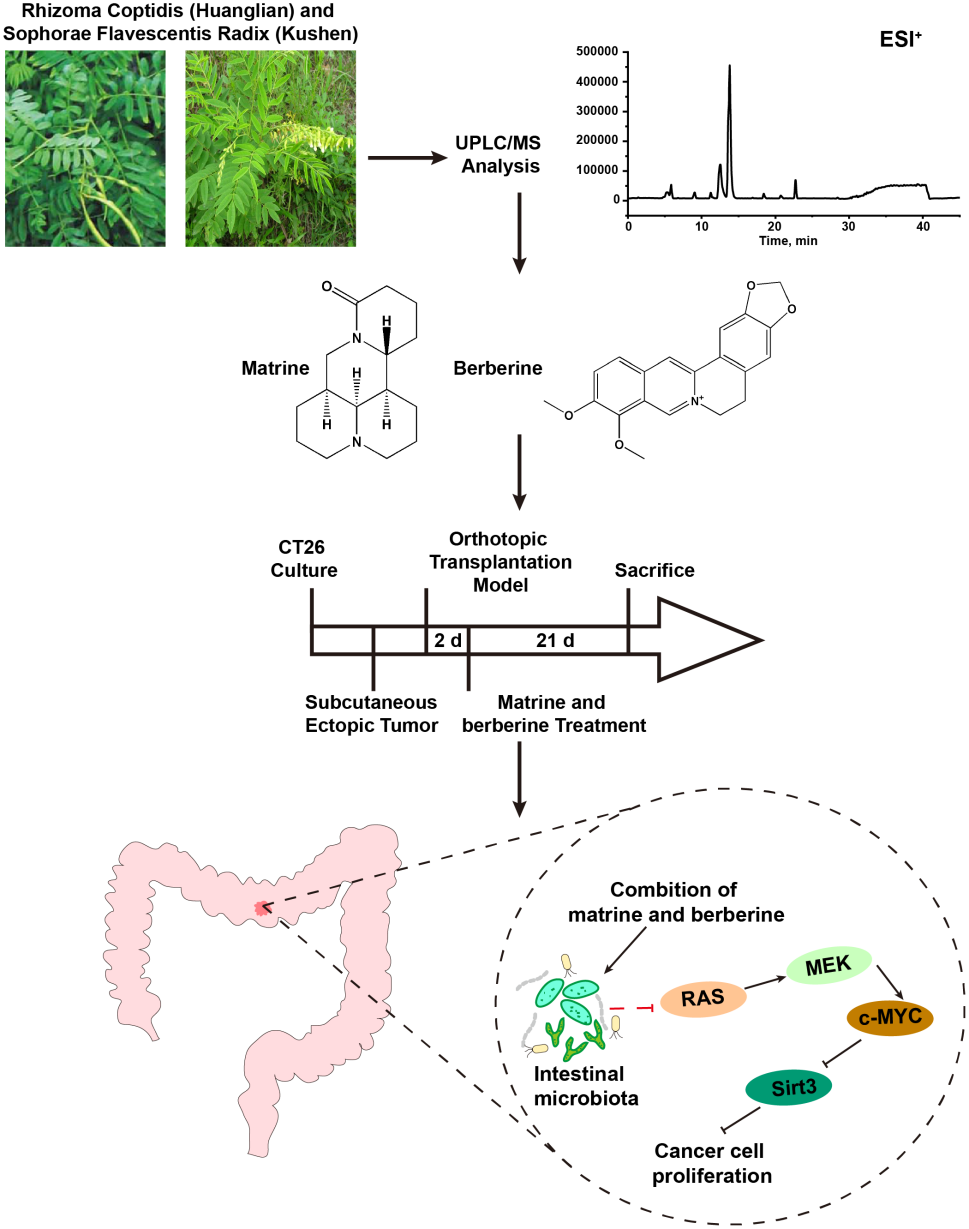
Mechanism underlying the anti-colorectal cancer effect of matrine and berberine.

Collectively, the results indicated that matrine and berberine may act against colorectal cancer by regulating the intestinal microecological and the signaling pathway related to cell proliferation (**Figure 6**). The regulating effect of matrine and berberine on RAS/MEK/ERK-c-MYC-Sirt3 signaling axis may be attributed to their influence on the composition of intestinal microbiota. The present research study may provide a reference for the use of matrine and berberine in the prevention and treatment of colorectal cancer. However, the mechanisms underlying these have not been investigated far. Hence, further studies are warranted to clarify the role and mechanism of intestinal flora in anti-colorectal cancer effects.

## Conclusion

The combination of Kushen and Huanglian at a1:1 ratio exerted the best anti-colorectal cancer effect among the combinations examined in this study. The chemical compounds contained in the Kushen and Huanglian extract were identified and evaluated. Additionally, the combination of matrine and berberine induced an almost equivalent anti-colorectal cancer effect to that noted after treatment with the drug pair. In addition, the combination exerted a better effect on colorectal cancer than monotherapy. The beneficial action of this treatment might depend on the improvement of the intestinal microbiota structure and regulation of the RAS/MEK/ERK-c-MYC-Sirt3 signaling pathway.

## Data availability statement

The datasets presented in this study can be found in online repositories. The names of the repository/repositories and accession number(s) can be found below: https://www.ncbi.nlm.nih.gov/, PRJNA866510.

## Ethics statement

The animal study was reviewed and approved by Nanjing University of Chinese Medicine Institutional Animal Care and Use Committee.

## Author contributions

DS, HC and JG conceived and designed the experiment. ZC, YD and QY collaborated in the experiments, analyzed the data, interpreted the results, and developed the manuscript. QL, LL, CY, YL and JT collaborated in the experiment and analyzed the data. MF, CX and WS supervised the work and proofread the manuscript. All authors contributed to the article and approved the submitted version.
